# Glutathione deficiency induces epigenetic alterations of vitamin D metabolism genes in the livers of high-fat diet-fed obese mice

**DOI:** 10.1038/s41598-019-51377-5

**Published:** 2019-10-15

**Authors:** Rajesh Parsanathan, Sushil K. Jain

**Affiliations:** Department of Pediatrics and Center for Cardiovascular Diseases and Sciences, Louisiana State University Health Sciences Center-Shreveport, 1501 Kings Highway, Shreveport, LA 71130 USA

**Keywords:** Biomarkers, Risk factors

## Abstract

Obesity has been correlating with low levels of glutathione (GSH) and 25-hydroxyvitamin D3 (25(OH)VD_3_). The liver is the principal site for the 25(OH)VD_3_ biosynthesis. This study investigated whether GSH deficiency induces epigenetic alterations that impair Vitamin D (VD) metabolism genes in the livers of HFD-fed mice. The expression of the VD metabolism genes CYP2R1 and CYP27A1 (25-hydroxylase), CYP27B1 (1-α-hydroxylase), and vitamin D receptor (VDR) were downregulated in the livers of mice fed an HFD (GSH- deficient) compared with control diet-fed group. The expression of CYP24A1 (24-hydroxylase) was significantly increased, which catabolizes both 25(OH)VD_3_ and 1α,25-hydroxyvitaminD_3_. Gene-specific hypermethylation of 25-hydroxylase, 1-α-hydroxylase, and VDR, and hypomethylation of CYP24A1 was observed in HFD-fed mice. GSH deficiency induced in cultured hepatocytes caused an increase in oxidative stress and alterations in VD regulatory genes. Similarly, elevated global DNA methylation, Dnmt activity, and 5-methylcytosine but decreased Tet activity and 5-hydroxymethylcytosine were observed in the GSH-deficient hepatocytes and the liver of HFD-fed mice. Replenishment of GSH by its prodrugs treatment beneficially altered epigenetic enzymes, and VD-metabolism genes in hepatocytes. HFD-induces GSH deficiency and epigenetically alters VD-biosynthesis pathway genes. This provides a biochemical mechanism for the VD-deficiency and potential benefits of GSH treatment in reducing 25(OH)VD_3_-deficiency.

## Introduction

Epigenetic regulation of gene expression refers to chromatin-based mechanisms that do not introduce changes in the DNA sequence *per se* and is not necessarily heritable. Gene-expression regulated by epigenetic modifications, such as alter DNA accessibility and chromatin structure, histone modification, and DNA methylation^1,2^. Moreover, evidence has emerged that a link exists between glutathione (GSH) metabolism and the epigenetic regulation of redox phenomena^3,4^. GSH as a physiological antioxidant fundamentally involved in the maintenance of cellular redox homeostasis^5^. We recently demonstrated that GSH has a positive relationship with 25(OH)vitamin D_3_ (25(OH)VD_3_) in the blood of type 2 diabetic and obese subjects^6–9^. Also, supplementation with L‐cysteine (LC), a rate-limiting precursor of GSH^5^, boosts the levels of GSH, reduces oxidative stress, and improves circulating 25(OH)VD_3_ levels^7–12^.

The liver is the principal site for the hydroxylation of cholecalciferol at carbon 25 by 25-hydroxylase enzymes (CYP2R1 and CYP27A1) to form 25(OH)VD_3_. The renal or extrarenal expression of 1-α-hydroxylase (CYP27B1) enzymatic action converts 25(OH)VD_3_ to an active metabolite 1α,25-dihydroxy vitamin D_3_ (1α,25(OH)_2_VD_3)_^13^. CYP24A1, a gene that provides instructions for making the enzyme 24-hydroxylase, is involved in the catabolism of both 25(OH)VD_3_ and 1α,25(OH)_2_D_3,_ thereby limiting vitamin D receptor (VDR)/1,25(OH)_2_D_3_ signaling^14^. The bioavailability of 25(OH)VD_3_ in the blood in response to dietary VD intake varies significantly among individual subjects and is dependent on the status of the VD metabolism genes^14–17^. This study examined the hypothesis that GSH-deficiency induces epigenetic alterations of VD metabolism genes, which can reduce the circulating 25(OH)VD_3_ levels in obesity.

## Results

### Impact of HFD on circulating plasma 25(OH)VD3 and GSH

The HFD-fed mice (16 weeks) gained more weight compared to standard chow diet-fed mice; the delta values calculated from the initial and final values collected during the HFD period of 16 weeks were significantly higher in HFD group. Blood glucose and fasting insulin levels were markedly elevated in HFD-fed mice and showed a higher HOMA insulin resistance index (Fig. S1A–D). This metabolic phenotype was similar to that of obese human type 2 diabetic subjects^18^. Plasma GSH and 25(OH)VD_3_ levels were significantly lower in HFD-fed animals compared to those in controls (Fig. S1E,F). Previous studies have shown a positive association between blood levels of 25(OH)VD and GSH in healthy adults and diabetic patients^8,19^. These findings are exciting because antioxidant molecule glutathione correlates with the measurable form of vitamin D. This led us to investigate whether impaired GSH status fuels 25(OH)VD_3_ deficiency/inadequacy epigenetically.

### HFD impairs liver glutathione biosynthesis, vitamin D metabolism genes and genes associated with non-alcoholic fatty liver disease (NAFLD)

**Figure 1:**
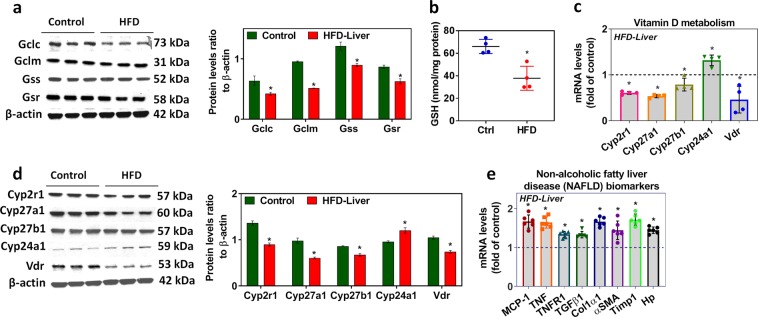
Effect of HFD on liver GSH and vitamin D metabolism genes. (**a**) Representative Western blot analysis (GCLC, GCLM, GSS, and GSR) performed on total protein extracts (n = 3) from the livers of mice fed an HFD for 16 weeks compared with those from mice fed the control diet. The *right panel* represents the semi-quantitative analysis of the protein abundance ratio to β-actin. **(b)** Liver GSH levels. **(c)** RT-qPCR was performed to assess the levels of vitamin D metabolism gene mRNA as indicated (n = 4). **(d)** Representative Western blot analysis (CYP2R1, CYP27A1, CYP27B1, CYP24A1, and VDR) performed on total protein extracts (n = 3) from the livers of mice fed an HFD for 16 weeks compared with those from mice fed the control diet. The *right panel* represents the semi-quantitative analysis of the protein abundance ratio to β-actin. **(e)** RT-qPCR was performed to assess the mRNA levels of genes (MCP-1, TNF, TNFR1, TGFβ1, Colα1, αSMA, Timp1, and Hp) associated with non-alcoholic fatty liver disease (NAFLD) as indicated (n = 6). An unpaired Student’s *t*-test was used to compare the control group with the HFD group. *p ≤ 0.05 was considered significant. Data are expressed as mean ± SEM.

Genes involved in the GSH biosynthesis pathway were significantly downregulated in the livers of mice fed an HFD compared to those of mice fed a healthy diet (controls) (Fig. S2A). The mRNA levels of liver GCLC and GCLM (Fig. S2A) and the protein levels of GCLC, GCLM, GSS, and GSR were significantly decreased in the HFD group (Fig. 1a). While the levels of GSH decreased significantly (Fig. 1b), those of oxidative stress markers such as protein carbonyl, reactive oxygen species, and lipid peroxidation were elevated in the livers of HFD-fed mice compared to those of controls (Fig. S2B,C,D). Additionally, the expression of mRNA and protein for both 25-hydroxylases (CYP2R1 and CYP27A1), 1-α-hydroxylase (CYP27B1), and VDR were downregulated, but that of 24-hydroxylase (CYP24A1) was significantly upregulated in the liver of HFD-fed mice compared to those in controls (Fig. 1c,d) which catabolize 25(OH)VD_3_ and active 1α,25(OH)_2_D_3_. The expression profile of genes monocyte chemoattractant protein-1 (MCP-1), tumor necrosis factor (TNF), tumor necrosis factor receptor type 1 (TNFR1), transforming growth factor-beta-1 (TGFβ1), collagen type I alpha 1 chain (Colα1,) actin alpha 2 smooth muscle (αSMA), tissue inhibitor of metalloproteinases 1 (Timp1), and haptoglobin (Hp) associated with non-alcoholic fatty liver disease (NAFLD) were elevated in the livers of mice fed an HFD for 16 weeks compared with those from mice fed the control diet (Fig. 1e).

### HFD alters epigenetic modifying enzymes and their activities in the liver

**Figure 2:**
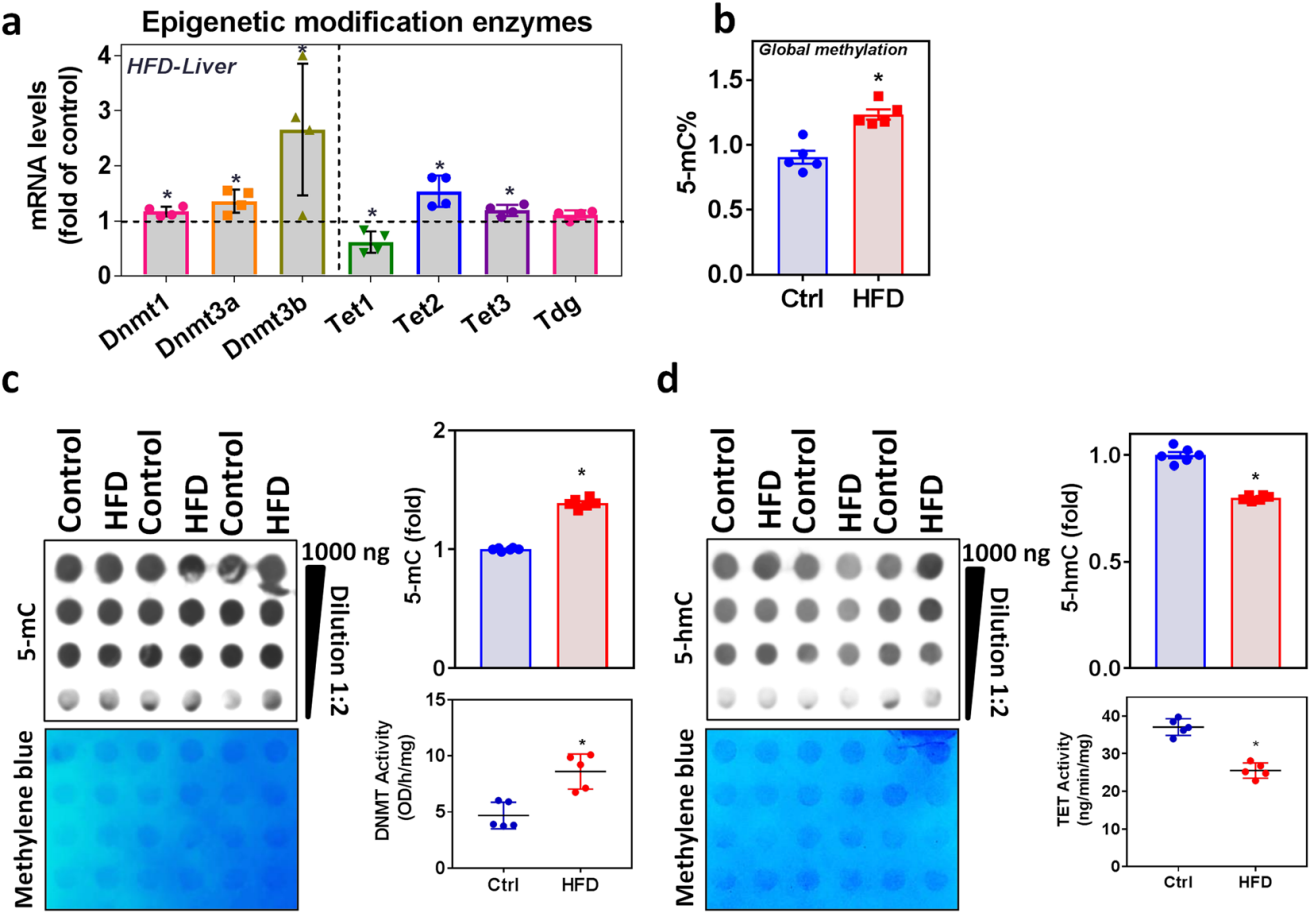
Effect of HFD on epigenetic modifying enzymes and its activities in the liver. (**a**) RT-qPCR was performed to assess the levels of the epigenetic modification enzyme mRNA (Dnmt1, Dnmt3a, Dnmt3b, Tet1, Tet2, Tet3, and Tdg) as indicated (n = 4). **(b)** Global methylation level (5-mC) determined using the ELISA method. **(c)** Semi-quantitative analysis of the dot-blot indicates 5-mC (fold) and the *left panel* shows a representative dot-blot of 5-mC (n = 6); liver nuclear extract Dnmt activity (n = 5). (**d**) Semi-quantitative analysis of the dot-blot indicates 5-hmC (fold) and the *left panel* shows a representative dot-blot of 5-hmC (n = 6); nuclear extract Tet activity (n = 5) from the livers of mice fed an HFD for 16 weeks compared with those from mice fed the control diet. An unpaired Student’s *t*-test was used to compare the control group with the HFD group. *p ≤ 0.05 was considered significant. Data are expressed as mean ± SEM.

DNA methylation and demethylation are the important epigenetic markers of gene regulation. The mRNA levels of the DNA methyltransferases 1, 3a, 3b, Tet2, and Tet3, were significantly upregulated in the liver of HFD-fed mice, whereas Tet1 was downregulated considerably (Fig. 2a). There was no change in Thymine-DNA glycosylase (Tdg) mRNA level. The global methylation status showed significantly increased 5-mC levels (Fig. 2b). Measurement of Dnmt activity showed an increased trend in HFD-fed mice liver, and Tet hydroxylase activity significantly inversed. DNA dot-blot analysis also in line with activity assay, which showed an increased 5-methylcytosine and decreased 5-hydroxymethylcytosine levels (Fig. 2c,d).

### HFD differentially methylates vitamin D metabolism enzyme genes and VDR

**Figure 3:**
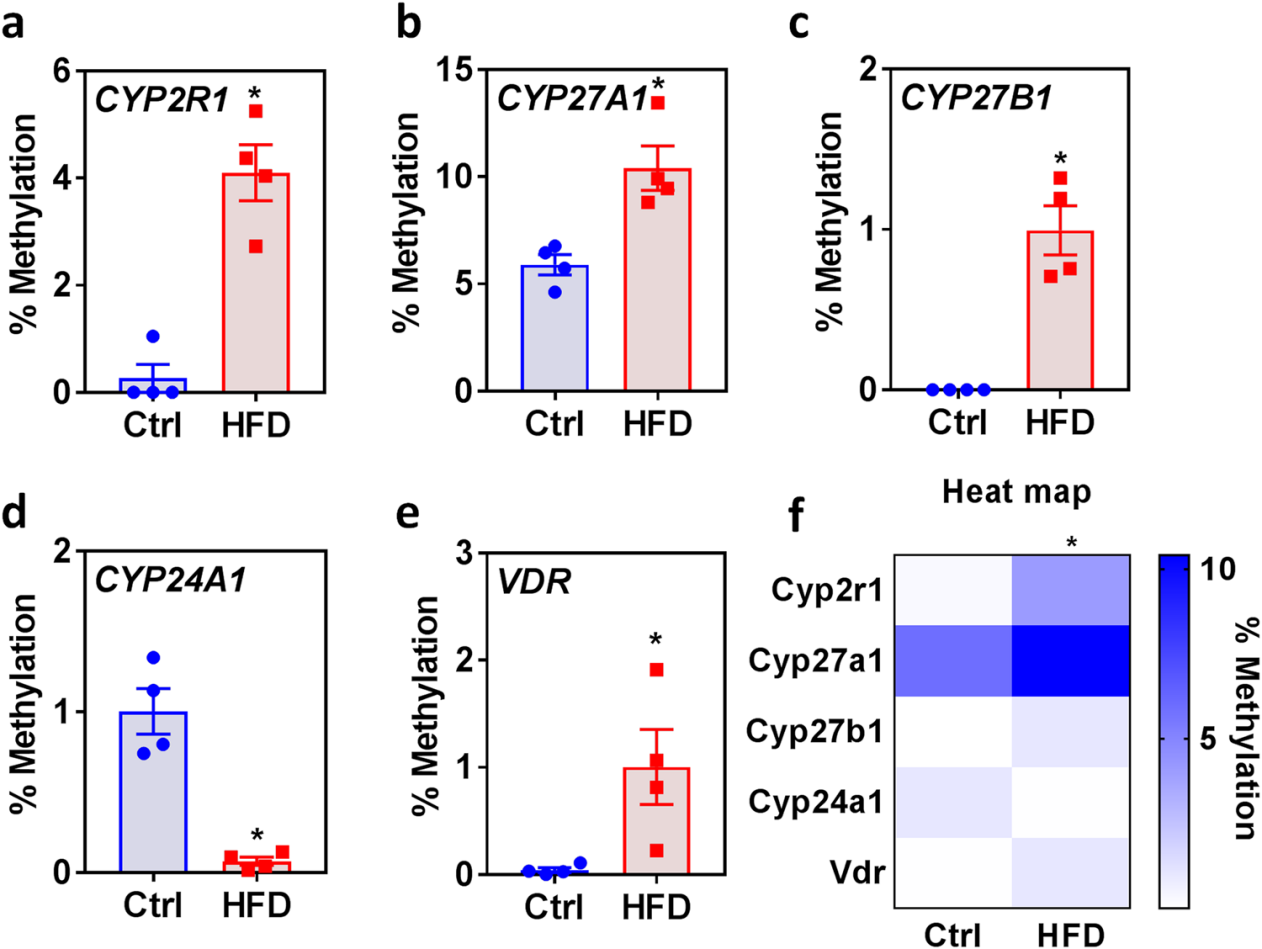
Effect of HFD on the methylation status of vitamin D metabolism enzyme genes in the liver. Percent methylation of each CpG island of vitamin D metabolism genes: **(a)** CYP2R1, **(b)** CYP27A1, **(c)** CYP27B1, **(d)** CYP24A1, and **(e)** VDR. **(f)** The heat map shows the vitamin D metabolism gene CpG island methylation (n = 4) in the genomic DNA from livers of mice fed an HFD for 16 weeks compared with those from mice fed the control diet, as determined using the EpiTect Methyl II PCR assay. An unpaired Student’s *t*-test was used to compare the control group with the HFD group. *p ≤ 0.05 was considered significant. Data are expressed as mean ± SEM.

To identify the gene-specific methylation of CpG island of vitamin D metabolism genes and VDR, we adapted a digestion-based gene-specific PCR methylation assay. Compared to livers from control mice, those from HFD-fed mice showed a significantly increased percentage of methylation at the CpG islands of CYP2R1 (Fig. 3a), CYP27A1 (Fig. 3b), CYP27B1 (Fig. 3c), and VDR (Fig. 3e). Surprisingly, the CYP24A1 methylation level (Fig. 3d) in the livers from HFD-fed mice was significantly lower than that in control livers. The heat map, shown in Fig. 3f; compares the vitamin D metabolism genes and VDR CpG island methylation in the genomic DNA of livers from HFD-fed mice with those of controls.

### *In vitro* glutathione deficiency alters vitamin D metabolism genes and VDR in mouse hepatocytes

**Figure 4:**
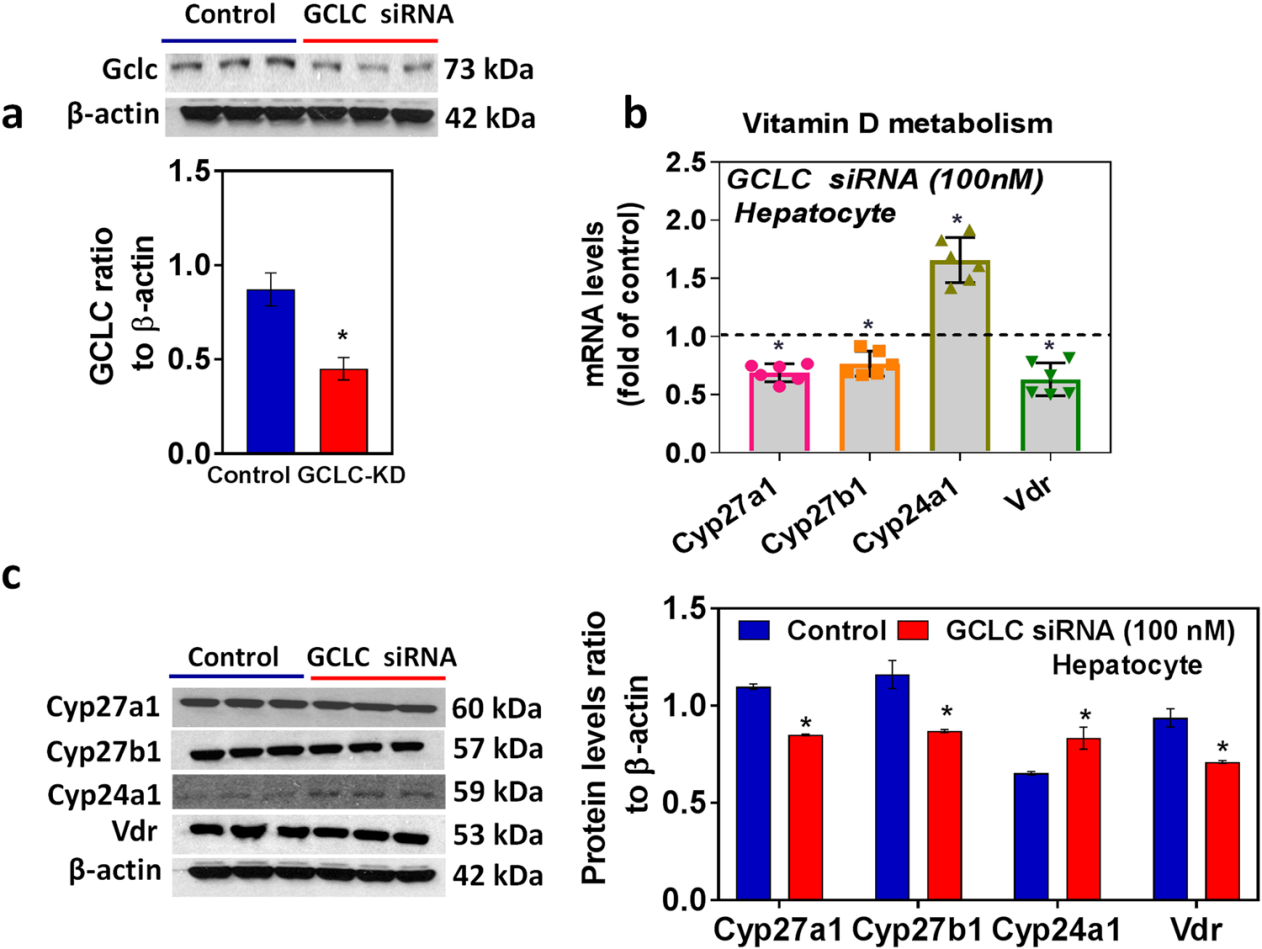
GCLC siRNA alters Vitamin D metabolism genes and VDR. FL83B mouse hepatocyte cells were incubated with siRNA (100 nM) targeting GCLC mRNA for 24 h. A scrambled non-targeting siRNA was used as the control. **(a)** Representative Western blot analysis of GCLC performed on total protein extracts (n = 3) and its semi-quantitative analysis of the protein abundance ratio to β-actin. **(b)** RT-qPCR was performed to assess the levels of vitamin D metabolism gene mRNA as indicated (n = 4). **(c)** Representative Western blot analysis (CYP27A1, CYP27B1, CYP24A1, and VDR) performed on total protein extracts (n = 3). The *right panel* represents the semi-quantitative analysis of the protein abundance ratio to β-actin. An unpaired Student’s *t*-test was used to compare the control group with the treatment group. *p ≤ 0.05 was considered significant. Data are expressed as mean ± SEM.

**Figure 5:**
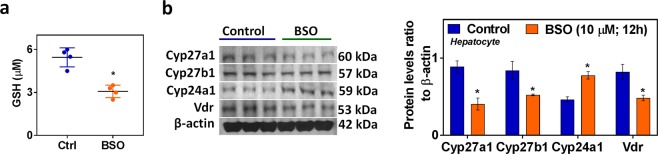
Effect of GSH deficiency (BSO, GCL inhibitor) on hepatocyte vitamin D metabolism genes. FL83B mouse hepatocyte cells were treated with a GCL pharmacological inhibitor (BSO) (10 μM) for 12 h. **(a)** GSH (n = 4). **(b)** Representative Western blot analysis (CYP27A1, CYP27B1, CYP24A1, and VDR) performed on total protein extracts (n = 3) of control and BSO treated groups. The *right panel* represents the semi-quantitative analysis of the protein abundance ratio to β-actin. An unpaired Student’s *t*-test was used to compare the control group with the treatment groups. *p ≤ 0.05 was considered significant. Data are expressed as mean ± SEM.

To rule out the non-specific effect of HFD conditions and other confounding variables in animals, we carried out *in vitro* experiments to determine the direct impact of glutathione deficiency status on VD metabolism genes in hepatocytes. GSH deficiency was induced by GCLC siRNA (Fig. 4a) or treatment with the pharmacological inhibitor BSO (Fig. 5a); in both approaches, cell viability was not affected in the present study. The mRNA and protein expression of the GSH biosynthesis pathway (GCLC, GCLM, GSS, and GSR) were downregulated in GCLC knockdown cells (Fig. S3A,B). Decreased levels of glutathione and elevated oxidative stress markers were observed in both siRNA (Fig. S3C–F) and inhibitor approaches (Fig. S4A–D). Under GSH-deficient conditions, treatment with siRNA (Fig. 4b,c) and the inhibitor BSO (Fig. 5b) resulted in decreased expression of CYP27A1, CYP27B1, and VDR. Both 25-hydroxylases (CYP2R1 and CYP27A1) were expressed in the liver, while CYP27A1 expression was seen only in cultured hepatocytes (FL83B mouse hepatocyte cell line). Additionally, CYP24A1 showed it upregulated mRNA and protein expression in hepatocytes treated with GCLC siRNA or its inhibitor. These results suggest that GSH may have a direct effect on VD metabolism.

### GSH deficiency on hepatocyte epigenetic modifying enzymes

**Figure 6:**
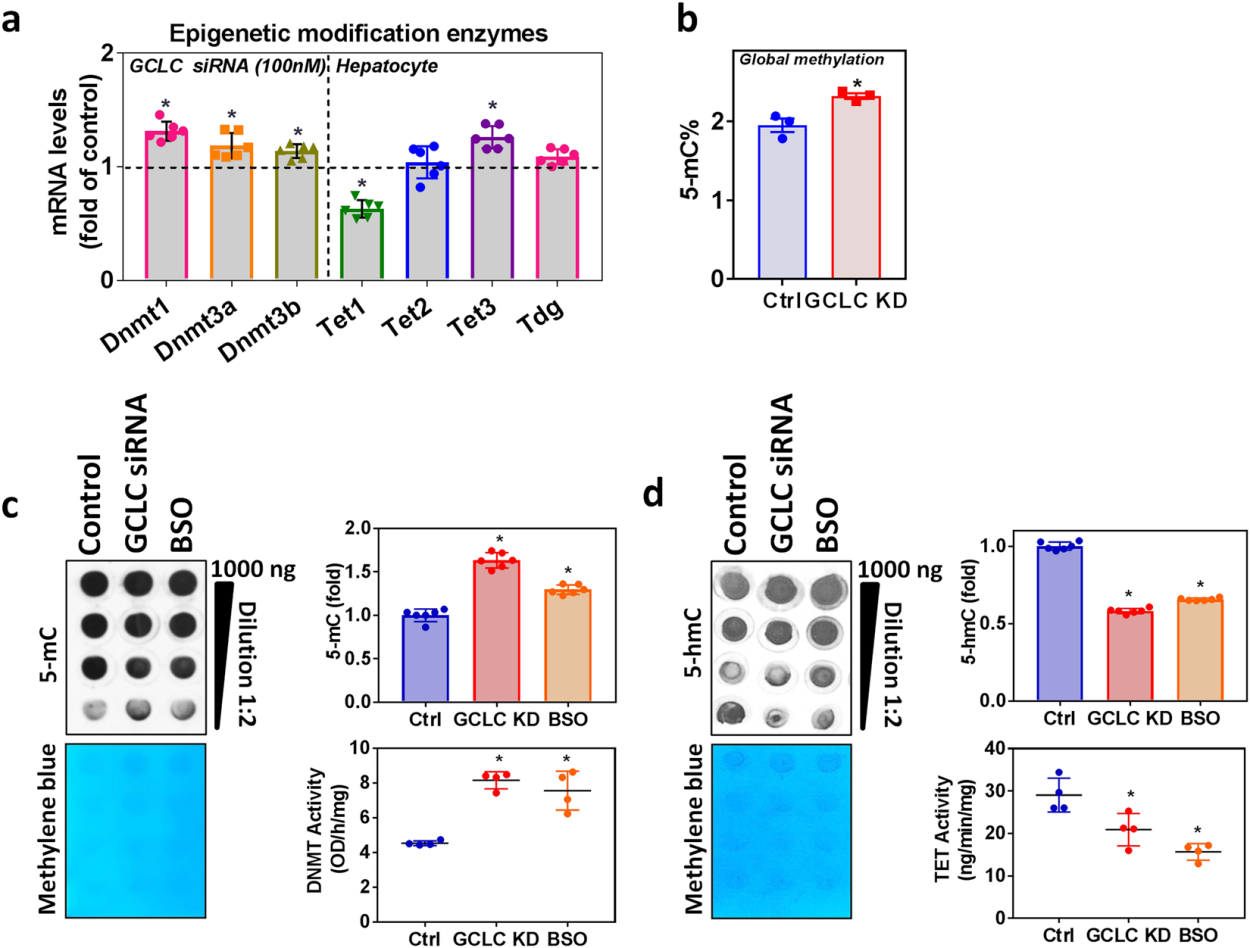
Effect of GSH deficiency (BSO, GCL inhibitor/siRNA) on hepatocyte epigenetic modifying enzymes and vitamin D metabolism genes. FL83B mouse hepatocyte cells were treated with a GCL pharmacological inhibitor (BSO) (10 μM) for 12 h or cells were incubated with siRNA (100 nM) targeting GCLC mRNA for 24 h. **(a)** RT-qPCR was performed to assess the levels of epigenetic modification enzyme mRNA (Dnmt1, Dnmt3a, Dnmt3b, Tet1, Tet2, Tet3, and Tdg) as indicated (n = 6) in GCLC siRNA cells. **(b)** Global methylation level (5-mC) determined using the ELISA method. **(c)** Semi-quantitative analysis of the dot-blot indicates 5-mC (fold) and the *left panel* shows a representative dot-blot of 5-mC (n = 6); hepatocyte nuclear extract Dnmt activity (n = 5). **(d)** Semi-quantitative analysis of the dot-blot indicates 5-hmC (fold) and the *left panel* shows a representative dot-blot of 5-hmC (n = 6); hepatocytes nuclear extract Tet activity (n = 5) in the GCLC siRNA and BSO treated groups. An unpaired Student’s *t*-test was used to compare the control group with the treatment groups. *p ≤ 0.05 was considered significant. Data are expressed as mean ± SEM.

Hepatocytes treated with GCLC siRNA showed elevated Dnmt, Tet2, and Tet3, but Tet1 was downregulated, and there was no change in Tdg mRNA expression (Fig. 6a). The global methylation status showed significantly increased 5-mC levels in GCLC knockdown cells (Fig. 6b); GSH-deficient cells showed increased Dnmt activity and decreased Tet hydroxylase activity (Fig. 6c,d). The DNA dot-blot showed the same trend in GSH deficiency and showed increased 5-mC levels with decreased 5-hmC levels (Fig. 6c,d). These data are in line with those from our animal experiments.

### Short-term BSO withdrawal effect on *in vitro* hepatocyte epigenetic modifying enzymes genes, vitamin D metabolism genes and genes associated NAFLD

**Figure 7:**
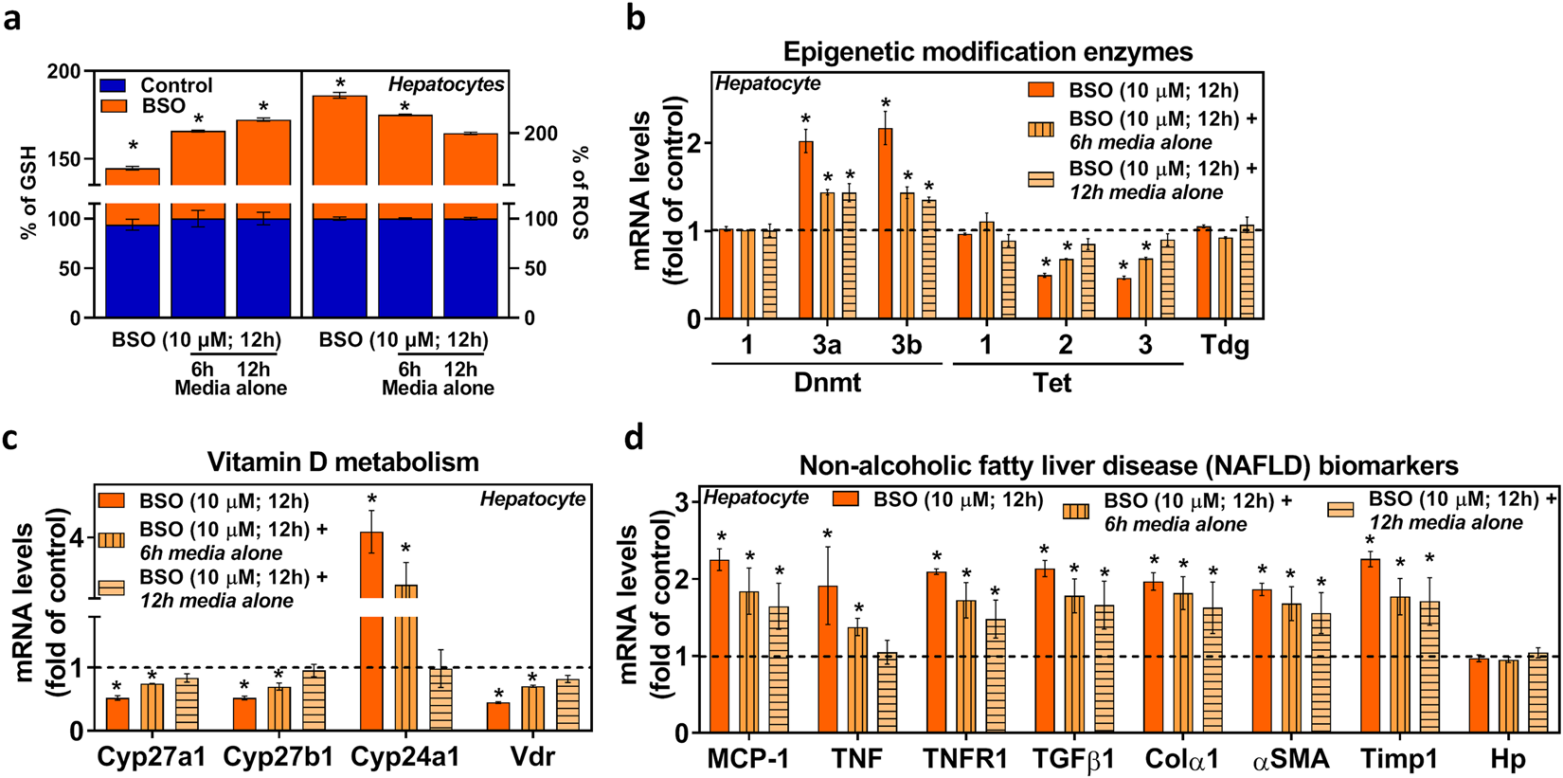
Effect of BSO withdrawal on hepatocyte epigenetic modifying enzymes genes, vitamin D metabolism genes, and genes associated with non-alcoholic fatty liver disease (NAFLD). FL83B mouse hepatocyte cells were treated with a GCL pharmacological inhibitor (BSO) (10 μM) for 12 h then cells washed twice with PBS and left either for 6 h or 12 h in basal media alone without BSO treatment (withdrawal). **(a)** GSH and ROS (n = 4). RT-qPCR was performed to assess the mRNA levels as indicated (n = 3). (**b**) Epigenetic modification enzyme genes. (**c**) Vitamin D metabolism genes. (**d**) Genes associated with NAFLD. An unpaired Student’s *t*-test was used to compare the control group with the treatment groups. *p ≤ 0.05 was considered significant. Data are expressed as mean ± SEM.

Treatment with BSO for 12 h showed a decline in GSH with an increased in ROS, whereas withdrawal of treatment for a period of 6 h and 12 h replenished the GSH content and decreased ROS in hepatocytes (Fig. 7a). To evaluate the short-term temporal dynamics of methylation and demethylation effects, we measured the expressions of epigenetic modifying enzyme genes. BSO treatment showed increased expression of Dnmt3a and 3b and decreased Tet2 and 3 transcripts, and it was not wholly restored after treatment withdrawal at different time points, as was shown by real-time PCR analyses (Fig. 7b). The similar trend was observed for vitamin D metabolism genes (Fig. 7c), suggesting the epigenetic association between GSH and vitamin D metabolism genes. Interestingly, GSH deficiency upregulates genes associated with NAFLD (Fig. 7d), and those transcripts persistently elevated even after BSO treatment withdrawal on both the time points. However, Haptoglobin (Hp) mRNA level was not altered due to BSO treatment.

### GSH and its precursors (L-cysteine and N-acetyl cysteine) treatments restore epigenetic modifying enzymes and VD metabolism genes

**Figure 8:**
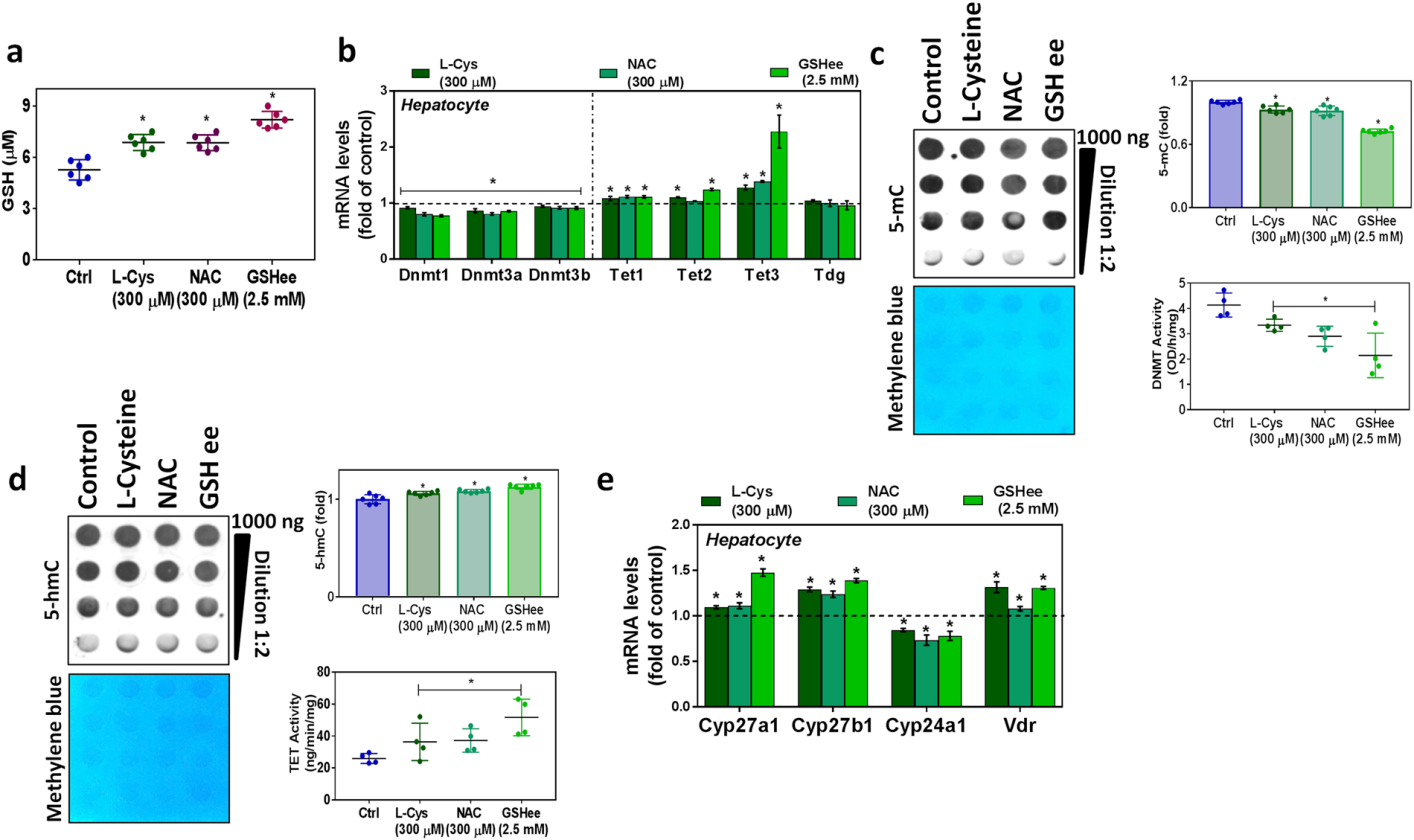
Effect of GSH and its precursors (L-cysteine and N-acetyl cysteine) on epigenetic modifying enzymes and vitamin D metabolism genes in hepatocytes. FL83B mouse hepatocyte cells were treated with L-cysteine, N-acetyl cysteine, or glutathione ethyl ester (soluble) as indicated. **(a)** GSH (n = 6). **(b)** RT-qPCR was performed to assess the levels of epigenetic modification enzyme mRNA (Dnmt1, Dnmt3a, Dnmt3b, Tet1, Tet2, Tet3, and Tdg). **(c)** Semi-quantitative analysis of the dot-blot indicates 5-mC (fold) and the *left panel* shows a representative dot-blot of 5-mC (n = 6); hepatocyte nuclear extract Dnmt activity (n = 4). **(d)** Semi-quantitative analysis of the dot-blot indicates 5-hmC (fold) and the *left panel* shows a representative dot-blot of 5-hmC (n = 6); hepatocytes nuclear extract Tet activity (n = 4) in the control and treated groups. (**e**) RT-qPCR was performed to assess mRNA levels of vitamin D metabolism genes (CYP27A1, CYP27B1, CYP24A1, and VDR) as indicated (n = 3). An unpaired Student’s *t*-test was used to compare the control group with the treatment groups. *p ≤ 0.05 was considered significant. Data are expressed as mean ± SEM.

Supplementation of hepatocytes with GSH or its precursors does not affect cell viability. The levels of GSH significantly increased after treatment with LC, NAC, and GSHee (Fig. 8a). There was a significant loss of Dnmt activity and rise in Tet activity in the treatment group. Data from DNA dot-blots and mRNA levels of Dnmt and Tet support the activity data (Fig. 8b–d). The levels of CYP27A1 and CYP27B1 were significantly upregulated along with those of VDR mRNA, but CYP24A1 was downregulated in groups supplemented with GSH or its precursor (Fig. 8e).

## Discussion

Deficiency of vitamin D (VD) is a significant and most common risk factor for chronic diseases, including insulin resistance, obesity, diabetes, and atherosclerosis^20–23^. Several studies reports impaired GSH and 25(OH)VD_3_ in obese and type 2 diabetic subjects^8,9,12,19,21^. This study also observed a decrease in circulating plasma GSH and 25(OH)VD_3_ in HFD-fed mice. The glutathione biosynthesis pathway activity was decreased in the livers of HFD-fed mice. Inflammation, steatosis, ballooning, and fibrosis are the features of non-alcoholic fatty liver disease (NAFLD), and the associated critical *bona fide* genes such as MCP-1, TNF, TNFR1, TGFβ1, Colα1, αSMA, Timp1, and Hp were significantly enriched in the livers of mice fed an HFD.

Furthermore, the levels of GSH decreased with increased oxidative stress markers in the HFD-fed mice, which reflect the exhaustion of antioxidant potential and increased oxidative stress. This suggests that GSH deficiency was initiated at the cellular level. Our study reports a link between decreased GSH status and impaired VD metabolism genes in the liver.

The present study observed reduced expression of 25-hydroxylase enzymes and 1-α-hydroxylase, vitamin D receptor, and increased expression of the 24-hydroxylase enzyme in the livers of HFD-fed mice. In the liver, the 25-hydroxylase enzymes (CYP2R1 and CYP27A1) convert inactive cholecalciferol to 25(OH)VD_3_ (calcidiol) by carbon 25 hydroxylation^14^. A study of humans with a genetic mutation for CYP2R1, or those done using CYP2R1 knockdown mouse models, demonstrate low circulating levels of 25(OH)VD_3_^24^. The regular vitamin D metabolic profiles illustrated with either wild-type mouse VDR or VDR null mice transgenic for the wild-type human VDR, but not the ligand binding-defective form of the protein, this suggests the essential roles of VDR on VD metabolism^25^. Also, our previous study showed that L-cysteine (LC) supplementation upregulates VDR and GSH status in the livers of Zucker diabetic fatty (ZDF) rats^7^. These results indicate that the loss of cellular glutathione and increase in oxidative stress alters vitamin D metabolism genes and vitamin D receptors, which can predispose the body to 25(OH)VD_3_ deficiency.

Epigenetic mechanisms are functionally essential for regulating gene expression^1,2^. Three DNA methyltransferase enzymes catalyze DNA methylation: Dnmt1, Dnmt3a, and Dnmt3b, which catalyze the transfer of a methyl group of S-adenosylmethionine (SAM) to the fifth carbon position of cytosine^26^. Ten-eleven Translocation (TET) enzymes converts methylcytosine (m^5^C) to hydroxymethylcytosine (hmC), which is not recognized by Dnmt1, it is believed to exclude maintenance methylation allowing for passive demethylation, and it is an intermediate in the active conversion of m^5^C to cytosine (C)^27^. A previous study showed that during the physiological cell cycle, there was a change in the nuclear GSH content^28^. Also, recent evidence suggests that GSH plays a crucial role in the control of epigenetic mechanisms^3,4,28–31^ either independently or by redox phenomenon.

The present study found increased DNA methyltransferases activity and gene expression of Dnmt1, 3a, 3b, and Tet (2 and 3). TET hydroxylase activity decreased along with Tet1 gene expression. DNA dot-blots for 5-mC and 5-hmC revealed the patterns created by the events of the epigenetic modification enzymes. We also examined whether expression of Dnmt contributes to global and gene-specific methylations, which alter the expression of vitamin D metabolism genes. The global DNA methylation in the livers of HFD-fed mice was increased, with significant gene-specific hypermethylation of CYP2R1, CYP27A1, CYP27B1, and VDR, and hypomethylation of CYP24A1. Global DNA hypermethylation is independent of established risk factors and associated with an increased risk of insulin resistance^31^. Evidence also exists that methylation memories in DNA are involved in maintaining gene expression patterns. Vitamin D signaling system genes, which has large CpG islands in their promoter regions and is prone to be regulated by DNA methylation and therefore can be silenced by *de novo* methylation^32–35^.

Functionally, DNA methylation is a repressive chromatin mark associated with gene silencing through a variety of different mechanisms; methylation either impeding the binding of transcription factors or by enriching the binding of methyl-binding proteins, which can sterically hinder *trans-* factor binding. Interestingly, recent studies have proposed that DNA methylation can lead to the exclusion of a histone variant (H2A.Z), preferentially located at active gene promoter^36,37^. These studies indicate that gene silencing correlates with DNA methylation, consistent with data from our study, which investigated vitamin D metabolizing gene expression. Our data suggest that hypermethylation of the 25-hydroxylases, 1-α-hydroxylase enzymes, and the vitamin D receptor promoter, accompanied by hypomethylation of CYP24A1, leads to impaired expression and possibly a predisposition to VD deficiency/inadequacy.

*In vitro* experiments have shown that GSH deficiency induced by either the siRNA approach or pharmacological inhibition of the GCL rate-limiting enzyme decreases GSH biosynthesis and escalates oxidative stress in cultured hepatocytes. Additionally, reduced expression of one 25-hydroxylase protein (CYP27A1; CYP2R1 expression was not seen in FL83B mouse hepatocytes) and 1-α-hydroxylase (CYP27B1), along with the increased expression of 24-hydroxylase (CYP24A1), was observed in GSH-deficient hepatocytes. These changes in expression may be due to decreased glutathione and increased oxidative stress; this rules out the non-specific effects of confounders in HFD-fed mice. Oxidative stress-induced formation of methionine sulfoxide can spontaneously react with hydroxyl radical and generates methyl radical that non-specifically and non-enzymatically methylate DNA cytosine and may affect the epigenome^38^. Also, it was shown that the epigenetic changes (DNA methylation patterns) to be associated with oxidative stress markers^39,40^. These observations suggest that altered expression of the VD metabolism genes is under the direct control of GSH.

To test the short-term temporal dynamics of methylation and demethylation effects, we measured the expressions of epigenetic modifying enzyme genes in BSO treatment withdrawal conditions in an *in vitro* mouse hepatocyte model. Interestingly, treatment withdrawal did not completely restore the mRNA transcripts, and a similar effect was observed for vitamin D metabolism genes and genes associated with NAFLD which was persistently higher, suggesting the epigenetic association between GSH deficiency and vitamin D metabolism genes.

This study suggests a link between GSH metabolism and the epigenetic regulation of VD metabolism gene. Dnmts and Tet enzymes mainly maintain DNA methylation and demethylation patterns^41^. In general, increased methylation results in lower gene expression. The importance and complex interplay of different epigenetic mechanisms play a vital role in gene expression. *In vitro*, GSH-deficient hepatocytes show upregulated mRNA levels of DNA methyltransferase, Tet2, and Tet3, along with increased Dnmt activity (5-mC) and global DNA methylation levels. In our study, we observed decreased levels of Tet1 mRNA and Tet hydroxylase activity. It has been shown previously that redox-dependent regulations of the TET isoforms^42^. Hence, GSH may interact with the epigenome on multiple levels.

Many antioxidant nutrients (folic acid, and vitamin B) and other dietary substances (green tea, and alcohol) are known to affect DNA methylation^43,44^. The modifications are reversible and are achieved with the aid of related enzymes. This study demonstrated that cultured hepatocytes treated either with a precursor of GSH, such as L-cysteine or N-acetylcysteine, or with its soluble ethyl ester form, boosts the levels of cellular GSH, which upregulates CYP27A1, CYP27B1, and VDR but downregulates CYP24A1. GSH replenishment in human tissues is difficult due to its shorter half-life, and not easy to penetrate the plasma membrane hence use of prodrugs/precursors such as cysteine, and glycine is the most promising approach^4,45^. This provides evidence for the positive effect of glutathione on VD metabolism and vitamin D receptor gene expression.

This study describes a novel biochemical mechanism by which glutathione deficiency causes epigenetic impairment of vitamin D metabolism gene in the liver and its contribution to reduced circulating levels of 25(OH)VD_3_ in HFD-fed obese mice. This study also suggests a potential role for GSH as an adjuvant therapeutic target for normalizing 25(OH)VD_3_ status in vulnerable populations.

## Materials and Methods

### Chemicals

All chemicals and reagents used in this study were of molecular and analytical grade and were purchased from Sigma Chemical Co. (St. Louis, MO) unless otherwise mentioned.

### Animals and diets

Male C57BL/6J mice (5 weeks old, 20–24 g) were purchased from The Jackson Laboratory (Bar Harbor, ME) and acclimatized in the Institutional Animal house for one week. Mice were divided into various groups by computer-generated randomization and then housed and labeled in individual cages. They were fasted overnight and then weighed. The mice were tested for hyperglycemia by measuring their blood glucose concentration. The animals were fed either a standard chow diet (Harlan TD.08485, providing 5.2% calories as fat; Control) or a high-fat diet (Harlan TD.88137, containing 42% calories as fat; HFD) for 16 weeks. The detailed composition of these diets appears in a recent publication^46^. The animals were maintained under standard housing conditions at 22 ± 2 °C with 12/12-h light/dark cycles. Bodyweight and blood glucose were monitored weekly. The amount of food intake was monitored at HFD period (16 weeks) to assess consumption and changes in body weight from baseline to post-intervention (HFD) was calculated. At the end of 16 weeks, the animals were fasted overnight and then euthanized by exposure to isoflurane (Webster Veterinary Supply Inc., Devens, MA). Blood was collected, from which plasma was isolated after centrifuging the blood in a 4 °C centrifuge at 2000 × *g* for 15 min; the plasma was stored at −80 °C until assays were performed. The animals were then perfused with cold saline to free them of residual blood, after which liver and other tissues were collected immediately, weighed, quickly diced, and frozen in liquid nitrogen at −80 °C. This model of dietary-induced insulin resistance created both fasting hyperglycemia and hyperinsulinemia and thus represented a reasonable model for the human condition. The study protocol was approved by the ethics committee of Louisiana State Health Sciences Center-Animal Care Committee (Animal Care protocol number P-15-006), and all the methods were carried out following the approved guidelines.

### Cell culture and treatment

FL83B mouse hepatocytes (ATCC^®^, Manassas, VA) were cultured and maintained in F-12K complete medium. Cell processing and maintenance of cell cultures were described previously^7,9^. Cell viability was determined using the Alamar Blue reduction bioassay^7,10,47^. Detailed information on the treatment is presented in the supporting file.

### 25(OH)VD, insulin, glucose, GSH, protein carbonyl, and Malondialdehyde (MDA) assays

Plasma levels of 25(OH)vitamin D were determined using an ELISA kit (Calbiotech, Spring Valley, CA), levels of insulin were determined using ELISA kits from ALPCO Diagnostics (Salem, NH); the HOMA insulin resistance index was calculated^48^. Protocols, as provided in the manufacturer’s instructions, were followed, including the use of appropriate controls and standards. Blood glucose was assessed using an Accu-Chek glucometer (Boehringer Mannheim Corp., Indianapolis, IN) with blood obtained via tail prick. Levels of GSH in plasma and tissues were determined using HPLC^47^, using an assay that determines total GSH status. GSH levels in cultured cells were quantified using a fluorimetric method (CS1020; Sigma, Saint Louis, Missouri) Oxidative stress was assessed by the quantification of protein carbonyls and MDA using Protein Carbonyl Colorimetric and TBARS Assay Kits, respectively (Cayman Chemical, Ann Arbor, MI).

### Gene expression and Western blot analysis

Total RNA was prepared from cells or tissue and reverse-transcribed. Relative expression of the indicated genes was measured with the Applied Biosystems™ TaqMan™ Gene Expression Assays with primer/probe sets (Supplementary Table S1), with Glyceraldehyde 3-phosphate dehydrogenase (GADPH) as a reference. Western blot analysis was performed as described previously^9^. Primary antibodies are detailed in Supplementary Table S2.

### Intracellular ROS production

Intracellular reactive oxygen species (ROS) levels were measured in treated cells using the oxidant-sensitive probe 2′,7′dichlorofluorescein diacetate [H_2_DCFDA]^11^. The change in intracellular ROS levels was plotted as mean fluorescence intensity (MFI).

### DNA methylation

Genomic DNA was isolated from the liver tissue or cultured cells using an AllPrep DNA/RNA/Protein Mini Kit (Qiagen, Germantown, MD) following the manufacturer’s instructions.

### Digestion-based DNA methylation analysis

Percentage DNA methylation was assessed at CpG sites across CpG islands in CYP2R1, CYP27A1, CYP27B1, CYP24A1, and VDR genes using Qiagen EpiTect II methylation enzyme kits and PCR assays at the concentrations and cycle conditions recommended by the manufacturer. Details of the CpG island size, location, and CpG content are described in the supplementary methods (Supplementary Table S3). This system uses selective digestion of sample DNA with methylation-sensitive and methylation-dependent restriction enzymes, followed by quantification of the remaining DNA using real-time PCR. Details of the reaction conditions, CpG sites assayed, and target site primers (Supplementary Table S3) are included in the supplementary methods.

### Global DNA methylation (5-mC) level

Levels of 5-methyl-2′-deoxycytidine (5-mC) were assessed in liver tissue and cultured cells using the 5-mC DNA ELISA Kit (Zymo Research, Irvine, CA). As per the manufacturer’s instructions purified genomic DNA was used (100 ng/well); detection: ≥0.5% 5-mC per 100 ng DNA. The percentage of 5-mC detected in DNA samples using a 5-mC DNA ELISA Kit strongly correlates with mass spectrometry (MS) data demonstrating 5-mC found in the same gDNA sample. The percentage of 5-mC was calculated using the second-order regression equation of the standard curve constructed with the negative and positive controls. Results are expressed as a percentage of 5-mC.

### DNA dot blot assay

The gDNA methylation level was detected using a dot blot assay as described previously with modifications^49^. In this dot blot approach, a gDNA to be detected was spotted directly onto a membrane as a dot, and then membranes blocked and probed with appropriated 5 mC or 5 hmC antibodies. Details of the antibodies and assay given in supporting information file.

### Dnmt and tet activity assays

The Dnmt activity and Tet activity assays were conducted using a Dnmt activity/inhibition assay kit (Epigentek, Farmingdale, NY) and a Tet activity/inhibition direct assay kit (Epigentek, Farmingdale, NY), respectively, following the manufacturer’s protocols. The nuclear protein fraction was extracted, and the relative Dnmt and 5-mC hydroxylase Tet activities were calculated based on the ratio of the treatment group to the control group after the normalization to the amount of protein.

### Statistics

The data were subjected to an unpaired Student’s *t*-test, which compares two averages (means), to assess the significance between results from control and experimental groups. The data are expressed as the mean ± standard error of the mean (SEM) and considered statistically significant at *p* < 0.05. All analyses were performed using GraphPad Prism 7 for Windows, version 7.04 (GraphPad Software, La Jolla, CA).

## Supplementary information

Supplementary Material
