# Supplementary material for: Glutathione deficiency induces epigenetic alterations of vitamin D metabolism genes in the livers of high-fat diet-fed obese mice
Source: Sci Rep. 2019 Oct 15;9:14784. doi: 10.1038/s41598-019-51377-5 (PMC6794254; doi:10.1038/s41598-019-51377-5)
Supplement: Supplementary file 1 — Supplementary Material [file 41598_2019_51377_MOESM1_ESM.pdf]

Online Supplementary information

Glutathione deficiency induces epigenetic alterations of vitamin D metabolism genes in the livers of high-fat diet-fed obese mice

Rajesh Parsanathan[1] and Sushil K. Jain[1]*

From the [1]Department of Pediatrics and Center for Cardiovascular Diseases and Sciences, Louisiana State University Health Sciences Center-Shreveport, 1501 Kings Highway, Shreveport LA 71130

*To whom correspondence should be addressed: Sushil K. Jain: Department of Pediatrics, 1501 Kings Highway, Shreveport, Louisiana 71130;
sjain@lsuhsc.edu: Tel. (318) 675-6086; FAX. (318) 675-6059

Contents

Cell culture and treatment 2
Cell viability assay 2
Analysis of mRNA expression using RT-qPCR 2
Supplementary Table S1: List of FAM-labeled TaqMan® primer/probe sets used for quantitative RT-PCR analysis 3
Western blot analysis 4
  Supplementary Table S2: List of antibodies and its dilutions used for Western blot analysis 4
DNA dot-blot assay 5
EpiTect II qPCR assays 5
  Supplementary Table S3: Details of CpG islands targeted by EpiTect Methyl II PCR Primer Assay for Mouse 6
Figure S1. 7
Figure S2. 8
Figure S3. 9
Figure S4. 10
References 10

Cell culture and treatment

FL83B mouse hepatocytes (ATCC®, Manassas, VA) were cultured and maintained in F-12K complete medium. The culture was grown and maintained at 37 °C in a humidified atmosphere containing 5% CO_2. Cells were counted using the Trypan Blue method before all treatments. siRNA were purchased from Santa Cruz Biotechnology, Inc. (Dallas, TX): γ-GCSc siRNA, and Control siRNA-A, a scrambled nonspecific RNA duplex that shares no sequence homology with any of the genes, which was used as a negative control. Cells were transiently transfected with 100 nM siRNA complex using Lipofectamine™2000 transfection reagent (Invitrogen, Carlsbad, CA) following the method described earlier [1,2]. In another set of experiments, cells were treated with L-cysteine (300 μM), N-acetyl-L-cysteine (NAC; 300 μM), or GSH ethyl ester (GSHee; 2.5 mM) (Cayman Chemical Company, Ann Arbor, Michigan), respectively, for 24 h. Buthionine sulfoximine (BSO) is an irreversible inhibitor of γ-glutamylcysteine synthetase (K_i <100 μM), the rate-limiting enzyme for L-glutathione (GSH) synthesis, which depletes GSH [3,4]. Cells were exposed to BSO (10 μM) for 12 h in basal medium (without serum or any growth factors). In another set of experiments, after BSO (10 μM) for 12 h in basal medium, cells washed twice with PBS and left either for 6h or 12h in basal media alone without BSO treatment (withdrawal).

Cell viability assay

Cell viability was determined using the Alamar Blue reduction bioassay. This method is based upon Alamar Blue dye reduction by live cells. Briefly, cells were plated into 96 well plates after treatment per the above-described protocols, AlamarBlue® Cell Viability Reagent (ThermoFisher Scientific, Grand Island, NY) was added, and the cells were incubated at 37 °C in the dark for 4 h. Absorbance was read at 590 nm using a plate reader. Data are expressed as a percentage of viable cells.

Analysis of mRNA expression using RT-qPCR

Total RNA was extracted from cells or tissue using either an AllPrep DNA/RNA/Protein Mini Kit (Qiagen, Germantown, MD) or TRIzol Reagent (Invitrogen, Carlsbad, CA) following the manufacturer's instructions. The concentration and quality of the extracted RNA were determined on a NanoDrop spectrophotometer. RNA (1 μg) from each sample was reverse transcribed according to the manufacturer's instructions using a High Capacity RNA-To-cDNA kit (Applied Biosystems, Foster City, CA) to synthesize cDNA. RT-qPCR was performed using the Applied Biosystems™ TaqMan™ Gene Expression Assays with primer/probe sets (Supplementary Table 1). The relative amount of mRNA was calculated using the relative quantification (ΔΔCT) method. The relative amount of each mRNA was normalized to housekeeping gene Glyceraldehyde 3-phosphate dehydrogenase (GADPH). Following Minimum Information for Publication of Quantitative Real-Time PCR Experiments (MIQE) guidelines, all of our experiments included technical replicates (n=3) and biological replicates (n=4). Data were analyzed using the comparative CT method, and the fold change was calculated using the $2^{-\Delta\Delta CT}$ method [5] using a 7900HT Real-Time PCR system and software (Applied Biosystems). The results were expressed as relative quantification (RQ).

Supplementary Table S1: List of FAM-labeled TaqMan® primer/probe sets used for quantitative RT-PCR analysis

Gene symbol	Gene Name	Assay ID	Transcript variant	RefSeq	Translated Protein	Exon Boundary	Assay Location	Amplicon Length	Context sequence
GCLC	Glutamate cysteine ligase, catalytic subunit	Mm00802655_m1	1	NM_010295.2	NP_034425.1	12--13	**1666**	98	tcattcc gctgtccaag gttgacga
GCLM	Glutamate cysteine ligase, modifier subunit	Mm01324400_m1		NM_008129.4	NP_032155.1	5--6	**963**	87	gctgtatcag tgggcacagg taaaa
GSS	Glutathione synthetase	Mm00515065_m1	2	NM_001291111.1	NP_001278040.1	5--6	**600**	67	ctc agctaatgcg gtggtgctac tg
			1	NM_008180.2	NP_032206.1	6--7	**735**	67	ctcagcta atgcggtggt gctactg
GSR	Glutathione reductase	Mm00439154_m1		NM_010344.4	NP_034474.4	8--9	**1116**	65	ttgaagt tcacacaggt taaggaag
CYP2R1	Cytochrome P450, family 2, subfamily R, polypeptide 1	Mm01159413_m1	1	NM_177382.4	NP_796356.2	1--2	**238**	76	cgggt gtatggcgag attttcagtt
CYP27A1	Cytochrome P450, family 27, subfamily A, polypeptide 1	Mm00470430_m1		NM_024264.4	NP_077226.2	2--3	**503**	69	cctatgggat cttcatcgca caagg
CYP27B1	Cytochrome P450, family 27, subfamily B, polypeptide 1	Mm01165918_g1		NM_010009.2	NP_034139.2	4--5	**820**	73	atc agatgtttgc ctttgcccag ag
CYP24A1	Cytochrome P450, family 24, subfamily A, member 1	Mm00487244_m1		NM_009996.3	NP_034126.1	7--8	**1357**	99	agtgga gacgaccgca aacagcttg
VDR	Vitamin D receptor	Mm00437297_m1		NM_009504.4	NP_033530.2	3--4	**289**	95	gttt cttcaggcgg agcatgaagc g
Dnmt1	DNA methyltransferase (cytosine-5) 1	Mm01151063_m1	1	NM_001199431.1	NP_001186360.2	5--6	**708**	58	cttca cgaagggccc cactaaacgg
			3	NM_001199432.1	NP_001186361.1	5--6	**589**	58	cttc acgaagggcc ccactaaacg g
			4	NM_001199433.1	NP_001186362.1	5--6	**1214**	58	cttcacgaa gggccccact aaacgg
			2	NM_010066.4	NP_034196.5	5--6	**705**	58	cttcacga agggccccac taaacgg
Dnmt3a	DNA methyltransferase 3A	Mm00432881_m1	3	NM_001271753.1	NP_001258682.1	17-18	**2339**	58	acag aagcatatcc aggagtgggg c
			1	NM_007872.4	NP_031898.1	17-18	**2385**	58	acagaagc atatccagga gtggggc
			2	NM_153743.4	NP_714965.1	12--13	**1563**	58	acagaagcat atccaggagt ggggc
Dnmt3b	DNA methyltransferase 3B	Mm01240113_m1	2	NM_001003960.4	NP_001003960.2	19-20	**2518**	83	gattc ctggcatgta acccagtgat
			1	NM_001003961.4	NP_001003961.2	20-21	**2578**	83	gattc ctggcatgta acccagtgat
			4	NM_001003963.4	NP_001003963.2	20-21	**2578**	83	gattc ctggcatgta acccagtgat
			5	NM_001122997.2	NP_001116469.1	19-20	**2466**	83	gattcct ggcatgtaac ccagtgat
			6	NM_001271744.1	NP_001258673.1	19-20	**2463**	83	gattcctggc atgtaaccca gtgat
			7	NM_001271745.1	NP_001258674.1	18-19	**2403**	83	gattcctggc atgtaaccca gtgat
			8	NM_001271746.1	NP_001258675.1	19-20	**2463**	83	gattcctggc atgtaaccca gtgat
			9	NM_001271747.1	NP_001258676.1	18-19	**2403**	83	gattcctggc atgtaaccca gtgat
			3	NM_010068.5	NP_034198.3	19-20	**2518**	83	gattc ctggcatgta acccagtgat
Tet1	Tet methylcytosine dioxygenase 1	Mm01169087_m1	1	NM_001253857.1	NP_001240786.1	9--10	**4851**	63	tg cttatcaaaa tcaggtggaa tat
			2	NM_027384.1	NP_081660.1	8--9	**4755**	63	tgcttatc aaaatcaggt ggaatat
Tet2	Tet methylcytosine dioxygenase 2	Mm00524395_m1	1	NM_001040400.2	NP_001035490.2	7--8	**4310**	70	aca gtggtggtca ccctcaatag ag
Tet3	Tet methylcytosine dioxygenase 3	Mm00805756_m1	2	NM_183138.2	NP_898961.2	9--10	**3121**	74	tg cactgtggtc tgcaccctga cca
Tdg	Thymine DNA glycosylase	Mm02602088_g1	1	NM_011561.2	NP_035691.2	9--10	**1202**	92	t caaatgggct aacagctcac agtg
			2	NM_172552.3	NP_766140.2	9--10	**1301**	92	tc aaatgggcta acagctcaca gtg
MCP-1/Ccl2	Monocyte Chemoattractant Protein-1	Mm00441242_m1		NM_011333.3	NP_035463.1	1--2	**165**	74	ctcagcca gatgcagtta acgcccca
TNF	Tumor Necrosis Factor	Mm00443258_m1	2	NM_001278601.1	NP_001265530.1	1--2	**352**	81	c ccaaagggat gagaagttcc caaa
			1	NM_013693.3	NP_038721.1	1--2	**352**	81	c ccaaagggat gagaagttcc caaa
TNFR1	Tumor Necrosis Factor Receptor Type 1	Mm00441883_g1		NM_011609.4	NP_035739.2	9--10	**1364**	82	cctgacaat gcagaccttg cgattc
TGFβ1	Transforming Growth Factor Beta-1	Mm01178820_m1		NM_011577.1	NP_035707.1	5--6	**1728**	59	ctatt gcttcagctc cacagagaag
Colα1	Collagen Type I Alpha 1 Chain	Mm00801666_g1		NM_007742.3	NP_031768.2	49-50	**4071**	89	cg atggattccc gttcgagtac gga
αSMA/Acta2	Actin Alpha 2, Smooth Muscle	Mm00725412_s1		NM_007392.3	NP_031418.1	9--9	**1403**	95	tagccctggc ctagcaacac tgatt
Timp1	Tissue Inhibitor Of Metalloproteinases 1	Mm01341361_m1	1	NM_001044384.1	NP_001037849.1	5--6	**618**	100	ggctg tggggtgtgc acagtgtttc
			3	NM_001294280.2	NP_001281209.1	4--5	**538**	100	ggctg tggggtgtgc acagtgtttc
			2	NM_011593.2	NP_035723.2	5--6	**615**	100	ggctgtgg ggtgtgcaca gtgtttc
Hp	Haptoglobin	Mm00516884_m1	1	NM_017370.2	NP_059066.1	4--5	**424**	69	aatgtgagg cagtgtgtgg gaagcc
GAPDH	Glyceraldehyde-3-phosphate dehydrogenase	Mm99999915_g1	1	NM_001289726.1	NP_001276655.1	2--3	**117**	107	ggtgtg aacggatttg gccgtattg
			2	NM_008084.3	NP_032110.1	2--3	**265**	107	ggtgtgaa cggatttggc cgtattg

Western blot analysis

The tissue homogenates were processed for the immunoblotting studies. ∼100 mg of liver tissue was homogenized in RIPA buffer on ice using a rotor-stator to extract protein from the liver. RIPA buffer (50 mM Tris pH 8, 150 mM NaCl, 1% NP-40, 0.5% deoxycholic acid, and 0.1% SDS) was supplemented with protease and phosphatase inhibitors (1 mM PMSF, 5 μg/mL leupeptin, 2 μg/mL aprotinin, 1 mM EDTA, 10 mM NaF, and 1 mM NaVO4). For whole-cell extraction, after treatment, the hepatocytes (FL83B cells) were washed twice with ice-cold PBS and lysed in RIPA buffer. Lysates were then centrifuged for 10 min at 10,000 x *g* at 4 °C. Supernatants were collected and the protein concentrations determined using a Pierce BCA assay kit (Thermo Fisher Scientific, Rockford, IL) for Western blot analysis and HPLC assay. Equal amounts of proteins (20 μg) were separated on 10 % SDS-PAGE and transferred to a polyvinyl difluoride (PVDF) membrane. Membranes were blocked at room temperature for 2 h in a blocking buffer containing 1% BSA to prevent non-specific binding and then incubated with an appropriate primary antibody (Supplementary Table 2). The membranes were washed in TBS-T (50 mmol/L Tris–HCl, pH 7.6, 150 mmol/L NaCl, 0.1 % Tween 20) for 30 min and incubated with the appropriate HRP-conjugated secondary antibody (1:5000 dilution) for 2 h at room temperature. The protein bands were detected using ECL detection reagents (Thermo Scientific, Rockford, IL) and exposed to blue X-ray film (Phenix Research Products, Candler, NC). All of our immunoblot experiments included technical replicates (n=2) and biological replicates (n=4). Western blot scans were analyzed using ImageJ software (developed by Wayne Rasband, National Institutes of Health, Bethesda, MD; available at http://rsb.info.nih.gov/ij/index.html). Densitometry analyses of Western blots were normalized to β-actin (ratio).

Supplementary Table S2: List of antibodies and its dilutions used for Western blot analysis

**Antibody Designation**	**Type**	**Antibody dilution and incubation time**	**Molecular weight (kDa)**	**Supplier (Catalog #)**
GCLC	Rabbit Polyclonal	1:500, 4° C overnight	73	Abcam (ab53179)
GCLM	Rabbit Monoclonal	1:1000, 4° C overnight	31	Abcam (ab126704)
GSS	Rabbit Monoclonal	1:10000, 4° C overnight	52	Abcam (ab124811)
GSR	Rabbit Polyclonal	1:2000, 4° C overnight	58	Abcam (ab16801)
CYP2R1	Rabbit Polyclonal	1:1000, 4° C overnight	57	Abcam (ab79924)
CYP27A1	Rabbit Monoclonal	1:1000, 4° C overnight	60	Abcam (ab126785)
CYP27B1	Rabbit Polyclonal	1:1000, 4° C overnight	57	Abcam (ab95047)
CYP24A1	Rabbit Polyclonal	1:1000, 4° C overnight	59	Abcam (ab175976)
VDR	Rabbit Polyclonal	1:1000, 4° C overnight	53	Abcam (ab3508)
β-actin	Mouse Monoclonal - HRP	1:25000, 2 h RT	42	Abcam (ab49900)
Goat anti-Rabbit-HRP Secondary Antibody		1:4000, 2 h RT	NA	Millipore (12-348)

DNA dot-blot assay

Genomic DNA was sonicated to generate fragments with lengths between 200 and 500 bp, denatured in 2x DNA denaturing buffer (200 mM NaOH and 20 mM EDTA), incubated at 95 °C for 10 min, and then immediately chilled on ice for 5 min. The denatured DNA fragments were spotted onto a positively charged 0.2 μm nitrocellulose membrane (Bio-Rad, Hercules, California). Briefly, DNA spots were air-dried for 15 min and UV-crosslinked (20 s, 1200J/cm^2). Membranes were blocked in 1% BSA in TBS-T (50 mmol/L Tris–HCl, pH 7.6, 150 mmol/L NaCl, 0.1 % Tween 20) for 2 h at room temperature (RT). Afterward, the membranes were incubated with appropriate primary antibodies (5-methylcytosine (5-mC) (D3S2Z), rabbit mAb (CellSignaling #28692, Danvers, MA), 5-hydroxymethylcytosine (5-hmC) (HMC31), or mouse mAb (CellSignaling #51660, Danvers, MA)) diluted 1:1000 in 1% BSA in TBST for 4 °C overnight. The membranes were washed in TBS-T for 30 min and incubated with the appropriate HRP-conjugated secondary antibody (1:5000 dilution) for 2 h at room temperature. After a final wash step, the membranes were incubated in Clarity Western ECL substrate (Bio-Rad, Hercules, California), and then 5-mC and 5-hmC were visualized using chemiluminescence and exposed on blue X-ray film (Phenix Research Products, Candler, NC). The membrane was stained with Methylene Blue (0.02% Methylene Blue in 3 M sodium acetate) after immunoblotting to ensure the equal loading. The membranes were destained by swirling them in distilled water. Dot-blot scans were analyzed using ImageJ software (developed by Wayne Rasband, National Institutes of Health, Bethesda, MD; available at http://rsb.info.nih.gov/ij/index.html). Densitometry semi-quantitative data analyses of the dot-blots are represented by fold change.

EpiTect II qPCR assays

DNA methylation was assessed using EpiTect II qPCR assays. The EpiTect II qPCR assays use differential digestion of unmethylated and methylated DNA, using methylation-sensitive (Ms) and methylation-dependent (Md) restriction enzymes, which reduces the risk of false-positive results. Digestions were conducted as individual digests (Ms or Md), in combination (Ms+Md), and as mock digests in a SimpliAmp Thermal Cycler (Applied Biosystems), at 37 °C overnight (16 h), followed by a 20 minute incubation at 65 °C to inactive the enzymes, as recommended by the manufacturer. The DNA is remaining after digestion was quantified using qPCR (Applied Biosystems 7900HT Fast Real-Time PCR System; 384-well), using primers flanking the region of interest and RT^2 SYBR green fluor qPCR master mix (Qiagen). The PCR reactions were carried out using the following conditions: 10 min at 95 °C, followed by 40 cycles of 15 min at 97 °C and 1 min at 72 °C, as recommended by the manufacturer. The relative amounts of methylation were calculated using an Excel-based data analysis template provided by the manufacturer, using delta-Ct values. The mock digest was used for initial DNA input quantification, the Ms digest to quantify methylated DNA and the Md digest for quantifying unmethylated DNA. The Ms+Md digest was used to quantify the undigested amount of DNA as a background control. EpiTect Methyl II PCR methylation-sensitive and methylation-dependent digest

control assays were used to test the cutting efficiency of the restriction enzymes and to ensure reliable and reproducible results.

Supplementary Table S3: Details of CpG islands targeted by EpiTect Methyl II PCR Primer Assay for Mouse

Gene	CpG Island ID	CpG Island Location	TSS Position	TSS Orientation	Size	Qiagen (Catalog #)
CYP2R1	110354	Chr7: 121706083 - 121706583	121706486	Reverse	500	EPMM110354-1A
CYP27A1	100226	Chr1: 74760192 - 74760405	74760147	Forward	213	EPMM100226-1A
CYP27B1	101224	Chr10: 126486076 - 126486319	126485301	Forward	243	EPMM101224-1A
CYP24A1	106871	Chr2: 170322710 - 170323048	170321927	Reverse	338	EPMM106871-1A
VDR	104088	Chr15: 97738408 - 97739146	97738727	Reverse	738	EPMM104088-1A

Figure S1.

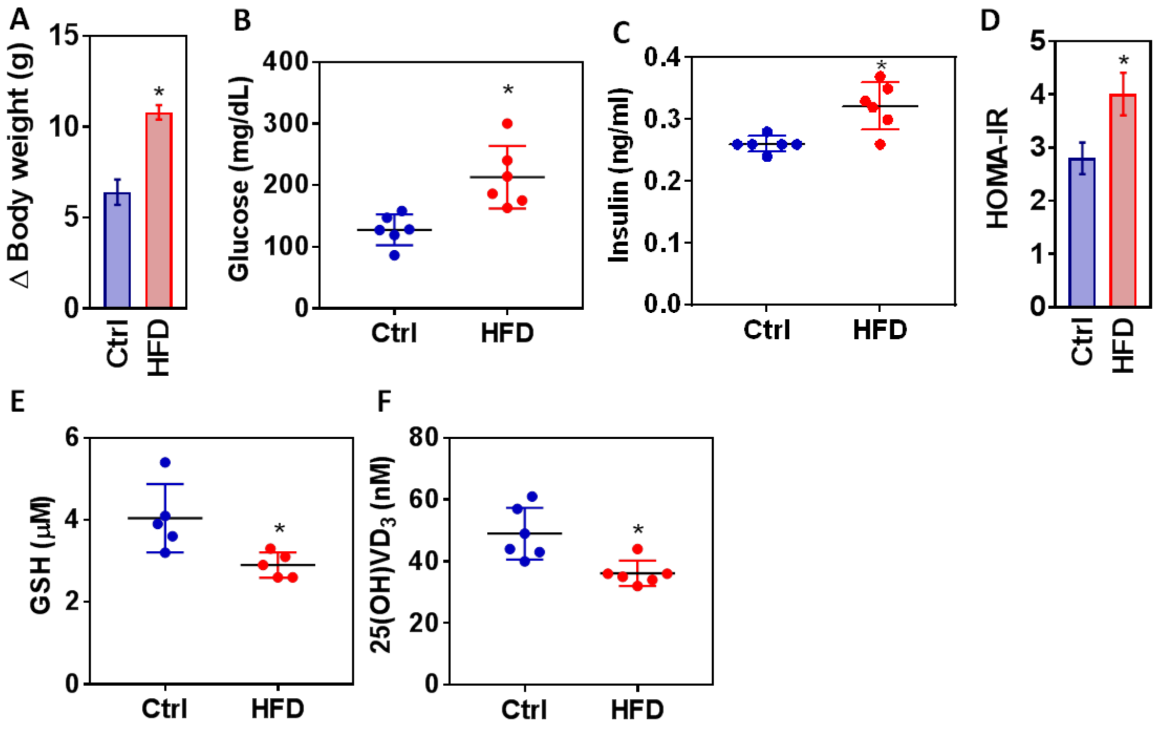

Figure S1. Impact of high-fat-diet on circulating plasma 25(OH)VD$_3$ and GSH. **(A)** Bar graph showing Δ body weight, **(B)** Blood glucose, **(C)** plasma insulin, **(D)** HOMA-Insulin Resistance, (**E**) plasma GSH, and **(F)** 25(OH)VD$_3$ in HFD-fed mice for 16 weeks compared with those of mice with the control diet. Unpaired Student's *t*-test was used to compare the control with the HFD group. *$p \leq 0.05$ for a statistical test was considered significant. Data are expressed as mean ± SEM (n=6**).**

Figure S2.

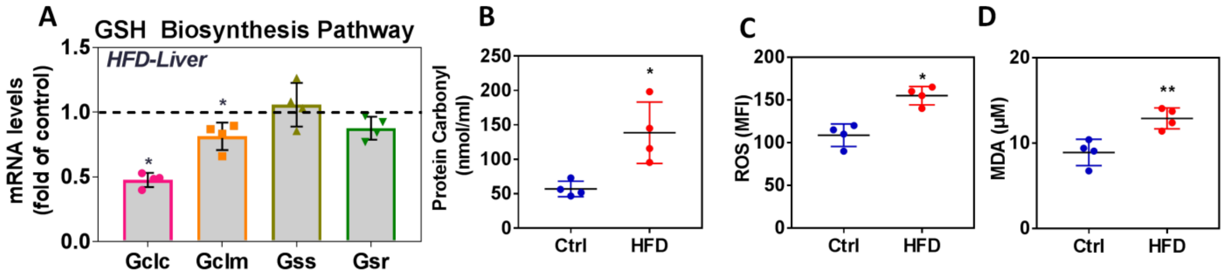

Figure S2. Effect of HFD on liver GSH biosynthesis pathway genes and oxidative stress parameters. (A) RT-qPCR was performed to assess the level of GSH biosynthesis in the pathway of the target gene mRNA as indicated (n=4). (B-D) Liver protein carbonyl content, reactive oxygen species, lipid peroxidation from the livers of mice fed an HFD for 16 weeks compared with those from mice fed the control diet. An unpaired Student's *t*-test was used to compare the control group with the HFD group. *$p \leq 0.05$ was considered significant. Data are expressed as mean ± SEM.

Figure S3.

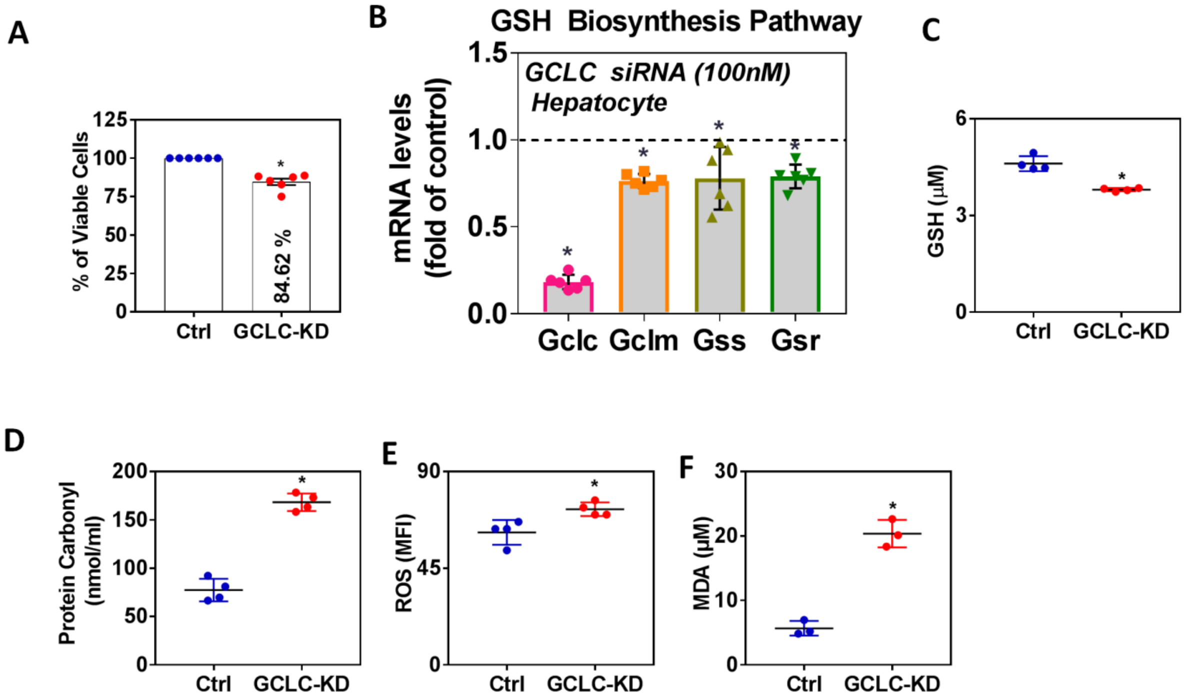

Figure S3. GCLC siRNA alters GSH biosynthesis pathway genes and oxidative stress parameters. FL83B mouse hepatocyte cells were incubated with siRNA (100 nM) targeting GCLC mRNA for 24 h. A scrambled non-targeting siRNA was used as the control. (A) Cell viability. (B) RT-qPCR was performed to assess the level of GSH biosynthesis in the pathway of the target gene mRNA as indicated (n=6). (C-F) GSH, protein carbonyl content, reactive oxygen species, lipid peroxidation. An unpaired Student's *t*-test was used to compare the control group with the treatment group. *$p \leq 0.05$ was considered significant. Data are expressed as mean ± SEM.

Figure S4.

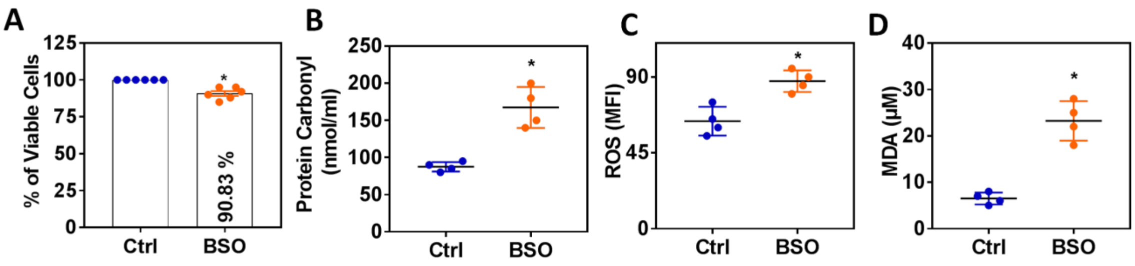

Figure S4. Effect of GSH deficiency (BSO) on hepatocyte oxidative stress parameters. FL83B mouse hepatocyte cells were treated with a GCL pharmacological inhibitor (BSO) (10 µM) for 12 h. (A) Cell viability. (B-D) Protein carbonyl content, reactive oxygen species, lipid peroxidation (n=4). An unpaired Student's *t*-test was used to compare the control group with the treatment groups. *$p \leq 0.05$ was considered significant. Data are expressed as mean ± SEM.

References

1 Parsanathan, R. & Jain, S. K. Hydrogen sulfide increases glutathione biosynthesis, and glucose uptake and utilisation in C2C12 mouse myotubes. *Free Radic Res* **52**, 288-303, doi:10.1080/10715762.2018.1431626 (2018).

2 Parsanathan, R. & Jain, S. K. L-Cysteine in vitro can restore cellular glutathione and inhibits the expression of cell adhesion molecules in G6PD-deficient monocytes. *Amino Acids* **50**, 909-921, doi:10.1007/s00726-018-2559-x (2018).

3 Reliene, R. & Schiestl, R. H. Glutathione depletion by buthionine sulfoximine induces DNA deletions in mice. *Carcinogenesis* **27**, 240-244, doi:bgi222 [pii]10.1093/carcin/bgi222 (2006).

4 Griffith, O. W. Mechanism of action, metabolism, and toxicity of buthionine sulfoximine and its higher homologs, potent inhibitors of glutathione synthesis. *J Biol Chem* **257**, 13704-13712 (1982).

5 Schmittgen, T. D. & Livak, K. J. Analyzing real-time PCR data by the comparative C(T) method. *Nat Protoc* **3**, 1101-1108 (2008).
